# Benign Tumors Associated With Heterozygous NTHL1 Variant

**DOI:** 10.7759/cureus.16220

**Published:** 2021-07-06

**Authors:** Danyon J Anderson, Andrew Boyle, Trenton Reinicke, Bison Woods, Patrick Hsieh

**Affiliations:** 1 School of Medicine, Medical College of Wisconsin, Wauwatosa, USA; 2 Department of Research, California Institute of Technology, Pasadena, USA; 3 Cancer Center, Massachusetts General Hospital Gastroenterology, Boston, USA; 4 Neurological Surgery, University of Southern California Keck School of Medicine, Los Angeles, USA

**Keywords:** nthl1, nthl1 tumor syndrome, peripheral schwannoma, schwannoma, hemangioma, genetic testing, tumor suppressor

## Abstract

*NTHL1* is a tumor suppressor gene involved in base excision repair. It is associated with an increased risk for colorectal and breast cancer when two variant gene copies are inherited. However, inheriting one variant *NTHL1* copy is not associated with increased tumor risk. Genetic counselors report heterozygous *NTHL1* mutations as benign. We present the case of a 22-year-old patient with a heterozygous *NTHL1* variant who developed an arm schwannoma, spinal schwannoma, and hepatic hemangioma. The patient also reported feeling multiple other bumps on his body but did not seek medical care due to a lack of symptoms. This case suggests that heterozygous *NTHL1* variants may be implicated in tumor development.

## Introduction

*NTHL1* tumor syndrome is a recessively inherited autosomal polyposis characterized by increased risk for colorectal polyposis, breast cancer, and colorectal carcinoma [1-5]. *NTHL1* is a tumor suppressor gene that acts through base excision to remove and replace damaged bases from DNA, preventing mutation [6,7]. Patients homozygous for *NTHL1* variants have been found to develop 14 different types of tumors affecting seven different organs [2]. *NTHL1* tumor syndrome is most famously associated with increased risk for colon cancer, breast cancer, and colorectal polyposis [2]. No research has been published yet on heterozygotes at increased risk for tumor formation. In heterozygotes, one functional copy of the *NTHL1* gene still facilitates proper base excision repair (BER). Regardless, there is a gap in the literature regarding the effect of being heterozygous for *NTHL1* variants. Here, we present the case of an *NTHL1* variant heterozygote who developed multiple benign tumors.

## Case presentation

**Figure 1:**
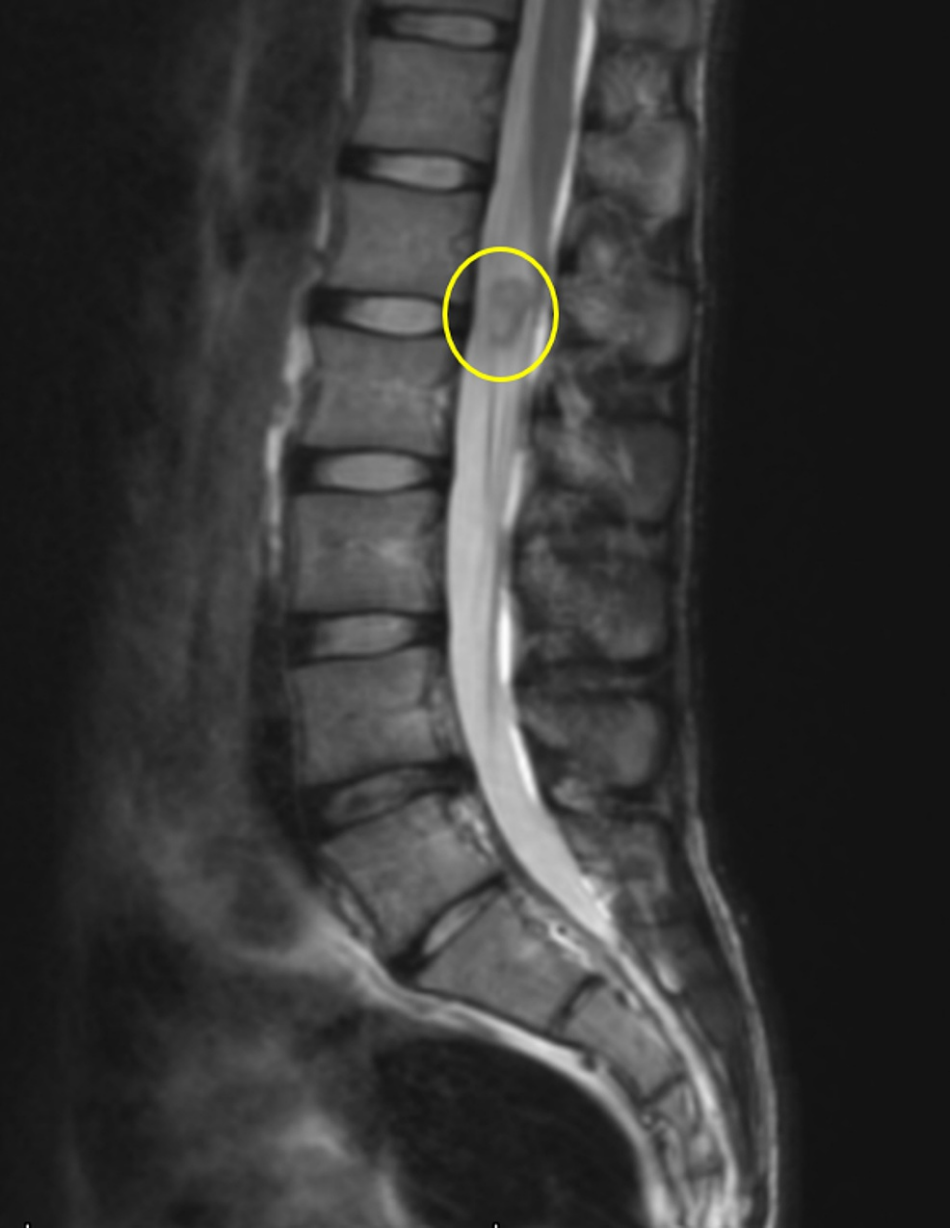
Possible schwannoma. A redemonstration of a 1.1 cm intradural extramedullary lesion at the L2-3 level within the left aspect of the thecal sac lesions in the adjacent cauda equina nerve roots favored to represent a schwannoma. This does not appear appreciably changed in size or appearance over the last year.

**Figure 2:**
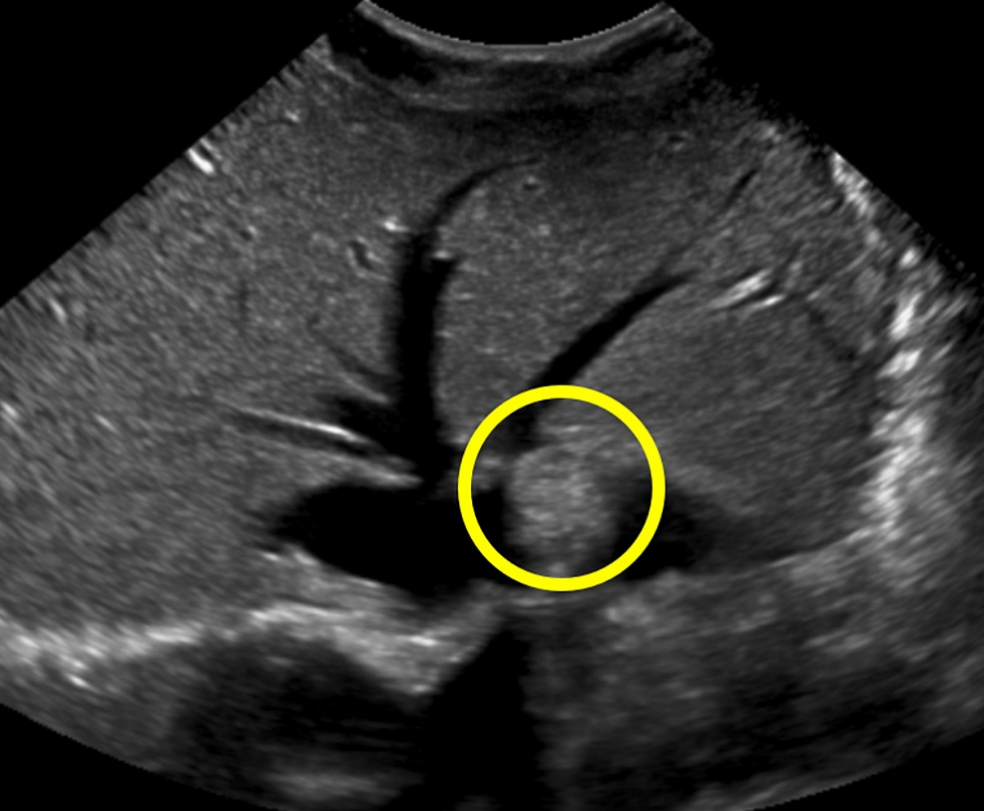
Hepatic hemangioma. A small 1.7 cm echogenic focus in the peripheral aspect of the left hepatic lobe, most compatible with an incidental hemangioma.

A 22-year-old white male with no significant past medical history presented to his primary care clinic two years ago for a bump on his left arm in the area of his biceps that caused intermittent pain in the distribution of the median nerve. The mass was excised and immunohistochemical staining determined it to be a benign schwannoma. A few months later, a tumor in the patient’s lumbar spinal canal was found incidentally during a hematuria workup. An MRI of the lumbar spine showed a tumor (Figure 1). The tumor had not grown from a CT scan two months prior, and although the tumor could not be biopsied, it appeared to be a schwannoma or myxopapillary ependymoma. The patient reported occasional low back pain and tingling down the left foot from this tumor. No other spinal tumors were seen on a full spinal MRI. An abdominal ultrasound evaluating symptoms of acid reflux found a 1.7 cm hemangioma in the left hepatic lobe and pericardial effusion (Figure 2).

At this point, the patient sought care to determine the cause of his tumors through genetic testing at the City of Hope. An* NTHL1* heterozygous variant was found. Tests were negative for variants in 86 other genes associated with cancer or tumors. At present, the patient reports feeling more bumps on his body but has not sought care due to a lack of symptoms.

## Discussion

DNA repair mechanisms are essential for maintaining efficiency and safety involved with genomic stability and proper cellular division. It is estimated that 30,000 damaged base lesions are repaired each day per cell by the BER pathway [8]. DNA glycosylases such as *NTHL1*, a bifunctional glycosylase, play a significant role in the BER pathway and act as the first line of defense to prevent genomic mutations caused by various environmental carcinogens such as mutagenic chemicals and distinct types of radiation [9]. Deficits in DNA damage repair pathways like BER are associated with the development of cancer, as well as a range of genetically inherited disorders, notably, *NTHL1*-associated polyposis (NAP) [4,8]. NAP is an autosomal recessive tumor syndrome characterized by aberrant functioning of the NTHL1 protein involved in DNA damage repair and is associated with a heightened risk for the development of colorectal cancer, breast cancer, and colorectal polyposis [1-5]. To our knowledge, the clinical diagnosis of the manifestation of multiple tumors due to having one mutant copy of *NTHL1* is not described in the literature.

This patient developed a schwannoma in his upper arm, a likely schwannoma in his lumbar spinal canal, a hemangioma in his liver, a pericardial effusion, and other bumps for which he did not seek care. It is natural to suspect that there is an underlying genetic cause for his tumors. He tested negative for variants in 86 of the most common tumor-associated genes and positive for a heterozygous variant in *NTHL1*. It is possible that this is the first published case of a benign tumor syndrome associated with a heterozygous *NTHL1* mutation. As *NTHL1* is a tumor suppressor gene, inheriting one defective copy would appear to increase the risk of developing tumors through sporadic loss of function of the other copy.

If these tumors did not result from his *NTHL1* mutation, it begs the question, what is the cause of these tumors if the other 86 tested genes involved in tumorigenesis were wild-type? Most nonidiopathic cases of three or more tumors in one patient are associated with a genetic cause [10,11]. Rare cases of multiple tumors can be linked to carcinogens such as tobacco or smoking [12-14]. However, this patient is a nonsmoker, is only 24 years old, and has not had excessive exposure to any known carcinogens. So, if his tumors are not due to his heterozygous *NTHL1* variation, they are likely due to mutation in a gene that was not one of the 87 tested. This may also be a sporadic case.

Future reports analyzing cases of multiple idiopathic tumors will be crucial in further understanding this patient’s condition. More cases showing multiple idiopathic tumors in patients with heterozygous variants in *NTHL1* would support our hypothesis that heterozygous *NTHL1* variants may cause a syndrome of multiple benign tumors, particularly schwannomas of peripheral nerves and hemangiomas of the liver.

## Conclusions

Heterozygous, in addition to homozygous, *NTHL1* variants may be associated with tumor development. Our presenting patient had a heterozygous *NTHL1* variant, with no other mutations in 87 tested genes, and he developed a spinal schwannoma, arm schwannoma, and hepatic hemangioma. To our knowledge, this is the first reported tumor syndrome in a patient that appears to be associated with a heterozygous variant of the *NTHL1* gene. Further investigation of heterozygous *NTHL1* variants will be necessary to understand its role in tumorigenesis.
